# Competing Anisotropy-Tunneling Correlation of the CoFeB/MgO Perpendicular Magnetic Tunnel Junction: An Electronic Approach

**DOI:** 10.1038/srep17169

**Published:** 2015-11-24

**Authors:** Chao-Yao Yang, Shu-Jui Chang, Min-Han Lee, Kuei-Hung Shen, Shan-Yi Yang, Horng-Ji Lin, Yuan-Chieh Tseng

**Affiliations:** 1 Department of Materials Science & Engineering, National Chiao Tung University, 1001 Ta Hsueh Road, Hsinchu, 30010, Taiwan, R.O.C; 2 Undergraduate Honors Program of Nano Science and Engineering, National Chiao Tung University, 1001 Ta Hsueh Road, Hsinchu, 30010, Taiwan, R.O.C; 3 Electronics and Optoelectronics Research Laboratories, Industrial Technology Research Institute, Hsin-Chu, Taiwan 30010, R.O.C; 4 National Synchrotron Radiation Research Center, Taiwan, 101 Hsin Ann Road, Hsinchu Science Park, Hsinchu 30076, Taiwan, R.O.C

## Abstract

We intensively investigate the physical principles regulating the tunneling magneto-resistance (TMR) and perpendicular magnetic anisotropy (PMA) of the CoFeB/MgO magnetic tunnel junction (MTJ) by means of angle-resolved x-ray magnetic spectroscopy. The angle-resolved capability was easily achieved, and it provided greater sensitivity to symmetry-related *d*-band occupation compared to traditional x-ray spectroscopy. This added degree of freedom successfully solved the unclear mechanism of this MTJ system renowned for controllable PMA and excellent TMR. As a surprising discovery, these two physical characteristics interact in a competing manner because of opposite band-filling preference in space-correlated symmetry of the 3*d*-orbital. An overlooked but harmful superparamagnetic phase resulting from magnetic inhomogeneity was also observed. This important finding reveals that simultaneously achieving fast switching and a high tunneling efficiency at an ultimate level is improbable for this MTJ system owing to its fundamental limit in physics. We suggest that the development of independent TMR and PMA mechanisms is critical towards a complementary relationship between the two physical characteristics, as well as the realization of superior performance, of this perpendicular MTJ. Furthermore, this study provides an easy approach to evaluate the futurity of any emerging spintronic candidates by electronically examining the relationship between their magnetic anisotropy and transport.

The combination of CoFeB and MgO is at the heart of advanced magnetic tunnel junction (MTJ) technology owing to the coexistence of excellent tunneling magneto-resistance (TMR) and strong perpendicular magnetic anisotropy (PMA). The prominent PMA can enable a reduction in current density and faster magnetization switching, which are particularly advantageous for spin-transfer-torque (STT) devices^1^. On the other hand, a coherent Δ1 channel^2^,^3^ developed by the CoFeB-MgO *p-d* hybridization can effectively tunnel spin electrons, yielding a giant TMR ratio for better signal identification. Owing to the broken symmetry at the CoFeB/MgO interface, a crystal field effect naturally develops with a complex spin-orbital coupling within the CoFeB^4^, which significantly alters the PMA and TMR by modifying the 3*d*-occupation of the degeneracy state. Recent studies had revealed that the *d*-band filling of the CoFeB with less *z*-correlated characteristics would give rise to PMA^5^,^6^. From a transport standpoint, an electronic channel with *z*-correlation would favor spin tunneling across the junction, according to Bloch theory^7^. These independent studies imply that though PMA and TMR share common characteristics in the orbital symmetry, a competing relationship may exist between the two physical characteristics owing to their opposite preferences in electronic occupation. We notice that in spite of continuous efforts devoted to the contact engineering of the CoFeB/MgO MTJ, its recent progress had come to a standstill after several groundbreaking works. This is because the two characteristics were often investigated independently^8^,^9^,^10^,^11^,^12^ and rarely considered in the same research setting. In this paper, we utilized angle-resolved x-ray magnetic spectroscopy to explore how the orbital symmetry electronically regulates TMR and PMA, following which a strict investigation of their cross interactions near the CoFeB/MgO hetero-junction was performed. This approach exclusively solved the unclear mechanism of the strong thickness-anisotropy sensitivity of CoFeB/MgO and successfully correlated it with TMR. To some extent, this approach is also novel in simultaneously predicting the MTJ’s TMR/PMA from an electronic perspective. We also discovered an overlooked superparamagnetic (SPM) phase hidden behind the MTJ’s signature PMA, which could be a fatal cause for the limited TMR. With a broader vision, the finding is beyond MTJ and equally important to heterostructured spintronic systems from the viewpoint of interfacial electronic effects in relation to the magnetic anisotropy and transport property.

## Results

**Figure 1:**
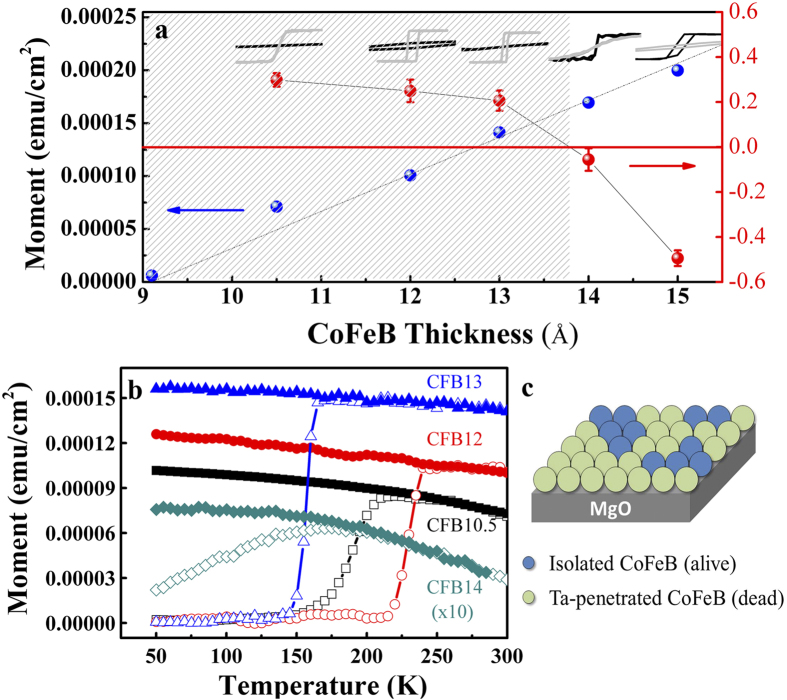
Thickness-dependent magnetic properties. (**a**) Thickness-dependent magnetic moment (blue balls, units on left y-axis) and effective anisotropy constant K_u_ (red balls, units on right y-axis) for the Ta (30 Å)/CoFeB (*t* = 9, 10.5, 12, 13, 14 and 15 Å)/MgO (11 Å)/Ta (100 Å)/Si multilayer structure. M-H curves corresponding to these five samples are superimposed at the top of the panel, where black and gray loops in each sample refer to in-plane and out-of-plane measurements, respectively. The gray area highlights the thickness regime with the PMA (positive K_u_). (**b**) Thickness-dependent M-T curves along the out-of-plane direction with ZFC (open symbol) and FC (filled symbol) conditions. (**c**) Illustration describing the discontinuous CoFeB magnetic phase isolated by Ta penetration on MgO surface.

Figure 1(a) presents the magnetic moment (blue-ball curve) as a function of CoFeB thickness for the Ta(30 Å)/CoFeB(9, 10.5, 12, 13, 14, and 15 Å)/MgO(11 Å)/Ta(100 Å)/Si multilayer sample. The CoFeB moment monotonically increases with increasing thickness, while a dead layer of 9 Å is found because of the zero moment obtained in the CoFeB = 9 Å sample. Anisotropy switching (indicated by the sign change of K_u_) from the out-of-plane to in-plane occurs with CoFeB thicknesses exceeding 13 Å, as supported by the thickness-dependent magnetic-hysteresis (M-H) curves superimposed on the top of Fig. 1(a). The critical thickness for anisotropy switching is consistent with classic references^8^,^9^, hence confirming our fabrication reliability. As revealed by the red-ball curve of Fig. 1(a), the anisotropy switching arises from a weakening of the out-of-plane anisotropy constant K_u_ upon increasing the thickness. Interestingly, despite the positive value of K_u_, the out-of-plane coercivity (H_c_) vanishes on reducing CoFeB to 10.5 Å, which suggests the loss of magnetic stability. A similar phenomenon has been reported by Cheng *et al.*^13^, wherein the same stacking structure exhibits a sharp drop of H_c_ around 10 Å. The zero-field-cooling (ZFC) and field-cooling (FC) measurements in Fig. 1(b) reveal that all the investigated samples intrinsically exhibit a blocking temperature (T_B_). This indicates the existence of a superparamagnetic (SPM) phase featuring short-range magnetic ordering. The SPM phase is ascribed to the 9-Å dead layer as a consequence of the inter-diffusion effect from the capping layer of Ta^14^,^15^. Upon annealing, Ta would penetrate CoFeB, causing demagnetization by destroying CoFe’s BCC structure. The penetrated Ta magnetically isolates CoFeB and causes it to behave as a uni-axial, single-domain Stoner–Wohlfarth particle on MgO’s surface^16^, which enables the SPM phase^17^,^18^. An illustration of the origin of SPM is given in Fig. 1(c), which refers to the coexistence of a major dead (~9 Å) and a minor alive CoFeB phase on MgO, at which the fraction of the alive phase is proportional to the CoFeB thickness exceeding 9 Å. This phenomenon of magnetic inhomogeneity (i.e., the coexistence of the dead and alive CoFeB) is validated by temperature-dependent H_c_ and x-ray magnetic spectroscopic sum-rule analysis (S-Fig. 1 of Supplementary Materials). Figure 1(c), in fact, rationalizes the irregular moment enhancement of CFB10.5 as a kind of magnetic instability because of the sudden formation of discontinuous but magnetically alive CoFeB phase spread over the 9-Å dead layer, which abruptly boosts the magnetization. Further growth of CoFeB magnetically stabilizes the film, as characterized by a perfectly linear moment–thickness dependency (Fig. 1(a)).

**Figure 2:**
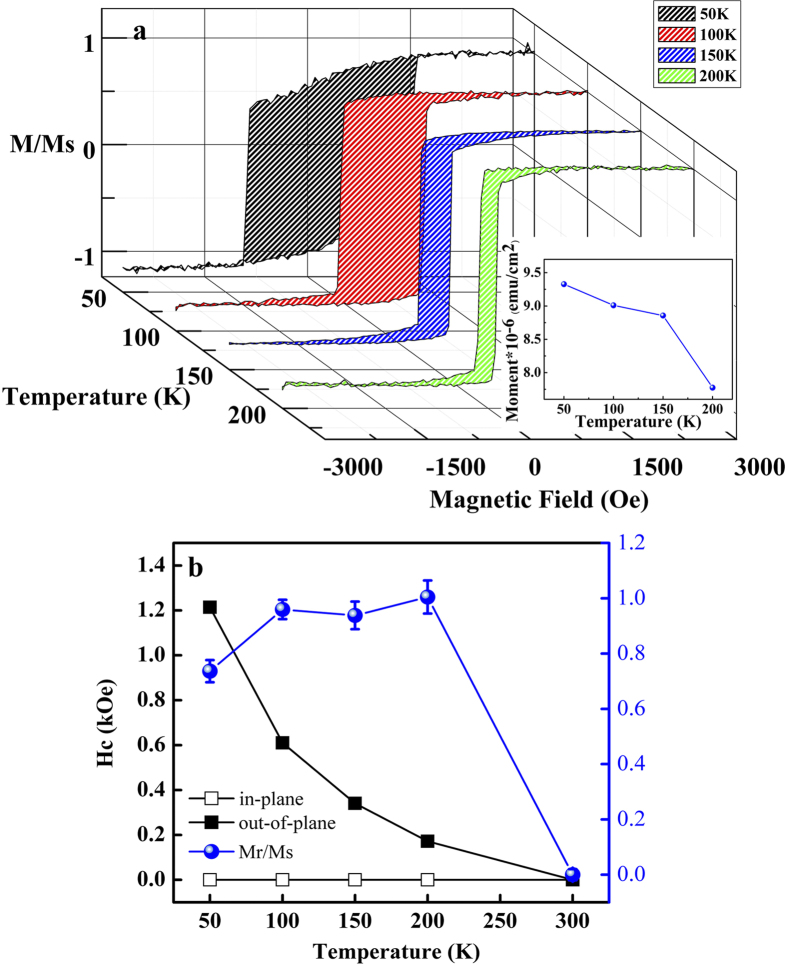
Temperature-dependent magnetic properties. (**a**) Temperature-dependent M-H curves of CFB10.5. Inset shows Ms as a function of temperature. (**b**) Temperature-dependent H_c_ (units on left y-axis) of CFB10.5 obtained from in-plane (open squares) and out-of-plane (filled squares) measurements. The magnetic squareness (M_r_/M_s_, units on right y-axis) of CFB10.5 obtained from out-of-plane measurements is presented by blue balls.

Despite better magnetic stability, SPM appears to be an inherent property because of the presence of T_B_ even if CoFeB exceeds 10.5 Å. This is essential to the MTJ technology, which relies on the CoFeB-MgO combination. In particular, this combination appears to have reached the TMR limitation in recent years, perhaps because of the harmful magnetic inhomogeneity. In fact, this phenomenon is easy to be overlooked by the CoFeB/MgO’s signature property of PMA. This is because magnetic anisotropy refers to a spatial moment-aligning preference, which is physically opposed to the SPM characterized by random flipping. To confirm this dual character, we performed temperature-dependent measurements on CFB10.5, the sample carrying the most significant SPM. In Fig. 2(a), the sample exhibits notable H_c_ at low temperature while gradually losing its magnetic stability upon warming. The saturation magnetization (M_s_) decreases by 10% from 50 K to 200 K (inset of Fig. 2(a)). This suggests a ferromagnetic (FM) → SPM transition due to thermal fluctuation. However, this magnetic transition is anisotropic because it only occurs out-of-plane, as indicated in Fig. 2(b). In addition, as an important indicator to magnetic anisotropy, the magnetic squareness (M_r_/M_s_, Fig. 2(b)) sharply drops out-of-plane for T > 200 K . This temperature coincides with CFB10.5’s T_B_ (200 K), at which SPM emerges (Fig. 1(b)). This implies that PMA is an overwhelming mechanism of CoFeB/MgO, which constrains the magnetic disordering effect (i.e., the SPM phase) to occur uni-axially.

**Figure 3:**
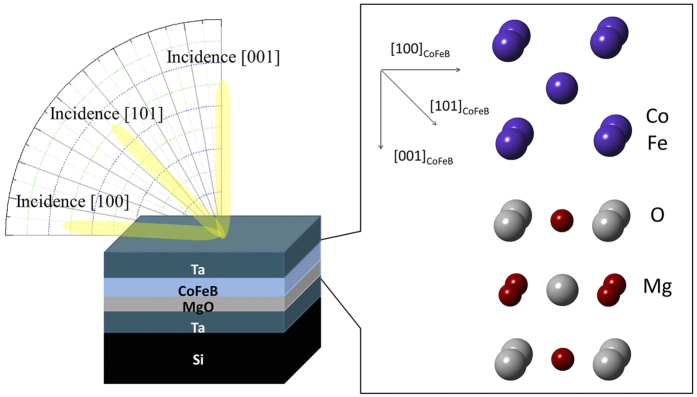
Angle-resolved XAS/XMCD measurements. (Left figure) Illustration showing angle-resolved XAS/XMCD performed on CoFeB/MgO, where three x-ray incident angles (5°, 45°, and 90°, with respect to the film’s plane) were chosen. The Miller indices ([001], [101], and [100]) of the x-ray incidences correspond to the three angles, with details given in the content. The three x-ray incidences independently probe the three crystallographic directions of CoFeB on MgO, with their geometric relations given in the right figure.

**Figure 4:**
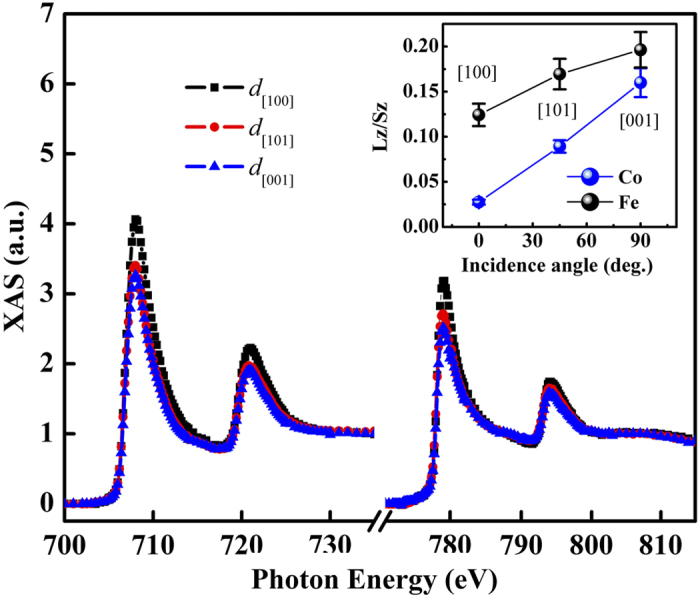
Angle-resolved XAS results. Angle-resolved *L*_*2*_*/L*_*3*_ XAS of Fe (left spectrum, photon energy from ~700 to ~730 eV) and Co (right spectrum, photon energy from ~770 to ~810 eV) taken from CFB10.5 at 300 K. The symbols of *d*[100], *d*[101], and *d*[001] correspond to the x-ray probing incidences of 5°, 45°, and 90° as described in Fig. 3. The inset presents the *L*_*z*_*/S*_*z*_ ratio of both Fe and Co with angle-dependency from XMCD sum rule analysis.

**Figure 5:**
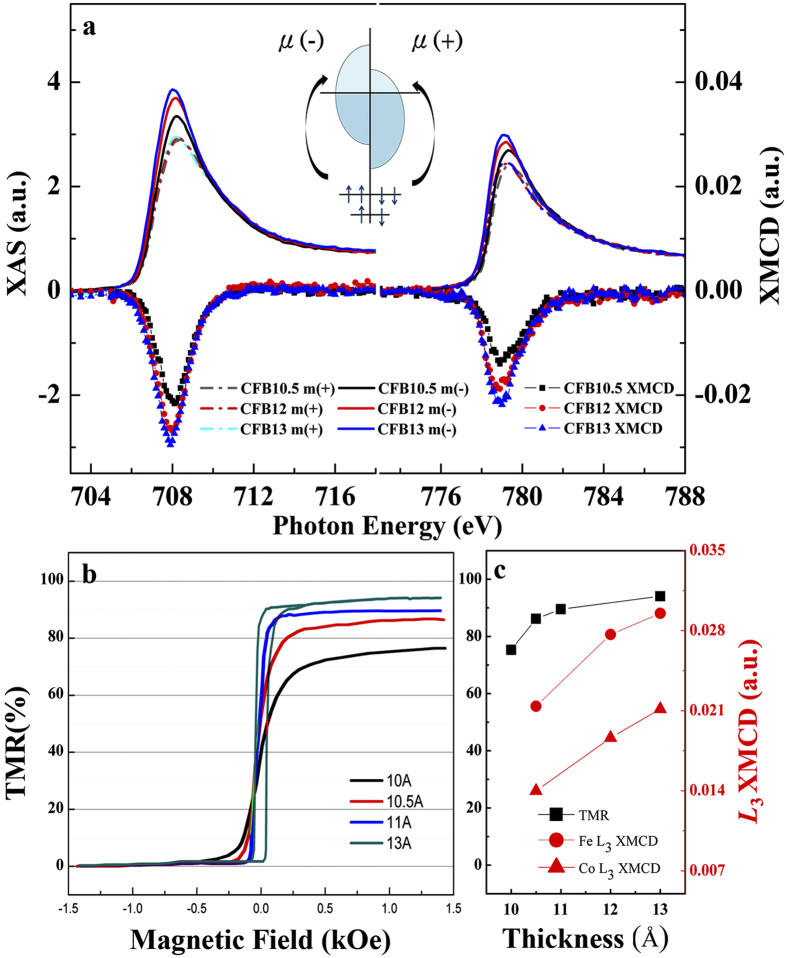
Thickness-dependent XMCD and TMR results. (**a**) Thickness-dependent *L*_*3*_ XAS of Fe (left spectrum) and Co (right spectrum) with positive (dashed line) and negative (solid lines) helicities. The corresponding XMCD obtained from the difference between two XAS helicities, together with thickness-dependency, are presented below the XAS spectra. *μ*(+) and *μ*(-) stand for the XAS probed by left and right x-ray helicities, respectively, whose intensities directly reflect the number of unoccupied majority and minority states, respectively, as illustrated in the middle inset of the figure. (**b**) The TMR (in %) for the MTJ with the CoFeB free layer of 10 Å, 10.5 Å, 11 Å, and 13 Å (bottom pinned CoFeB layer was fixed to 9 Å). The magnetic field range for TMR measurements was +/−1.5 kOe. The positive and zero values of the TMR refer to the antiparallel and parallel states of the two CoFeB electrodes in MTJ, respectively. (**d**) Comparison for the TMR and Fe/Co XMCD with respect to CoFeB thickness. The left y-axis unit of (**c**) shares the same TMR scale as that of (**b**).

Angle-resolved XAS/XMCD was then performed on the samples with three incident x-ray angles of 5°, 45°, and 90° with respect to the film’s in-plane direction, as depicted in Fig. 3. This is to explore the complex electronic effects that regulate the PMA. The collected angle-dependent Fe (left figure) and Co (right figure) *L*_*2*_*/L*_*3*_ XAS of CFB10.5 are presented in Fig. 4. Here, spectra collected from incident x-ray angles of 5°, 45°, and 90° correspond to the symbols of *d*[100], *d*[110], and *d*[001], respectively, which are crystallographically related to the 3*d* electronic configuration probed along the [100], [110], and [001] directions of the CoFeB described in Fig. 3, respectively. It is noteworthy that the Fe XAS intensity significantly decreases on rotating the film from the in-plane direction ([100]) to the out-of-plane direction ([001]). Since the XAS intensity is proportional to the number of unoccupied electronic states, this suggests that the electrons prefer to occupy the degeneracy-lift states of the out-of-plane *d*-band orbital (defined as *d*[001] henceforth). The angle-dependent sum-rule analysis from Fe/Co XAS/XMCD is presented in the inset of Fig. 4, where data of orbital-to-spin (*L*_*z*_*/S*_*z*_) moment ratio are given for the consideration of electronic-configuration calibration. We find that the *d*[001] occupation preference results in an increase of *L*_*z*_. An identical trend is observed in the angle-dependent Co *L*_*2*_*/L*_*3*_ XAS. The coherent enhancements of Co/Fe *L*_*z*_ as a result of the electronically filled *d*[001] could indicate the origin of PMA. In fact, theoretical studies^5^,^6^ have suggested that the PMA is driven by the crystal-field effect arising from the *d*-band degeneracy due to CoFeB/MgO’s broken symmetry at the interface; our result is the first that validates this hypothesis. For further validation, in Fig. 5(a), we collected helicity-dependent Fe and Co *L*_*2*_*/L*_*3*_ XAS of CFB12 and CFB13, in addition to CFB10.5, by fixing the x-ray to [001]. We define *μ*(+) and *μ*(-) as the XAS spectra with positive and negative helicities generated from circularly polarized x-rays, respectively. Therefore, *μ*(+) and *μ*(-) intensities are inversely proportional to the occupations of majority and minority spin states of *d*[001], respectively, based upon a spin-dependent photo-excitation process (middle inset of Fig. 5(a)). The minority states (*μ*(-) intensity) of Co and Fe appear to decrease coherently with increasing thickness. However, the majority states (*μ*(+) intensity) are independent of the thickness change, as a localized electronic characteristic of hard magnets^19^. On correlating with Fig. 2(a), where K_u_ is modified but the PMA is persistent in this thickness regime (10.5–13 Å, gray area of the figure), Fig. 5(a) suggests that the CoFeB/MgO electronically drives the PMA by coherently populating electrons in the *d*[001] minority states of the two magnetic elements. This substantiates the PMA-*d*[001] correlation from the viewpoint of the electronic spin state and thus assigns this correlation as the dominant mechanism for PMA. By thinning CFB13 to CFB10.5, the heterojunction’s broken-symmetry effect is intensified, leading to a more electronically filled *d*[001] that is responsible for the pronounced PMA. This finding constitutes the first experimental evidence for solving the elusive mechanism responsible for the strong thickness-anisotropy sensitivity of CoFeB/MgO, despite its extensive applications in MTJ over the years. Therefore, the controversial SPM-anisotropy phenomenon can be understood as a coincident nature where the *d*[001] occupation electronically/energetically forces CoFeB/MgO to align perpendicularly in spite of a secondary, short-range ordering effect arising from a magnetically inhomogeneous interface underneath (Fig. 2(c)).

Figure 5(a) also sheds lights on CoFeB’s spin-polarized states from Fe and Co *L*_*3*_ XMCD spectra probed along [001] with thickness dependency. The XMCD signal, originating from the difference between majority and minority occupations, is an indicator of the level of spin polarization of the chosen element. Given the fact that both Co’s and Fe’s XMCD signals are more optimized in CFB13 than in the other two samples, we understand that increasing the thickness (reducing broken symmetry) would enhance the spin polarization by unequally populating the *d*[001] spin states of Co and Fe. TMR, which is physically supported by the different tunneling possibilities for majority/minority electrons, is therefore expected to be reflected by XMCD. In other words, the XMCD intensity (i.e., spin polarization) of the *d*[001] would reveal TMR information because the spin-polarized *d*[001] state shares electronic similarities with the specific tunneling mechanism of CoFeB/MgO^2^,^3^,^7^. Following this principle, therefore, a higher TMR ratio should be achieved by increasing the CoFeB thickness, because of the enhanced spin polarization of the *d*[001] states, on the basis of the XMCD results. This hypothesis is exclusively supported by the thickness-dependent TMR data in Fig. 5(b), where a minute thickness increment of 0.5 Å in the CoFeB free layer can lead to notable TMR enhancement, and this TMR-enhancing trend persists up to 13 Å, where the PMA ends (Fig. 1(a)). This points to a strong sensitivity of the TMR to the *d*[001] spin polarization, which can actually be probed/predicted by XAS/XMCD.

## Discussion

**Figure 6:**
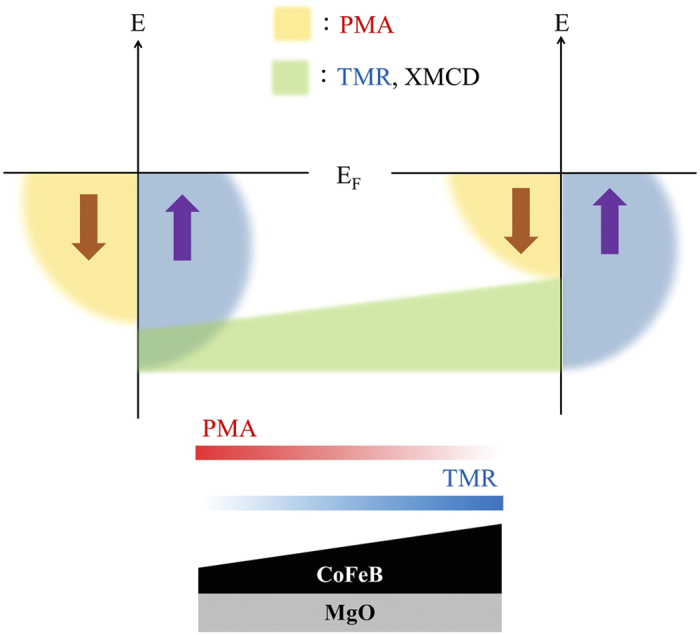
Spin-electronic diagram with respect to PMA and TMR. Thickness effects (bottom figure, with a thickness range of 10.5–13 Å) resulting from the evolution of the spin-electronic diagram of CoFeB (upper figure) on an MgO substrate. The electronic diagram is constructed based on the *d*[001] occupation probed by the angle-resolved XAS/XMCD. Yellow and blue parts in the electronic diagrams represent the occupied minority and majority states, respectively, and the transparent green bar linking the two diagrams corresponds to the change of minority occupation (while the majority is unchanged) that determines the TMR strength, which is reflected by XMCD. Gradient bars for PMA (red) and TMR (blue) indicate their strength changes with thickness and describe the opposite/competing correlation between the two physical characteristics.

To summarize the PMA-TMR relationship from XAS/XMCD results, we provide a spin-dependent electronic diagram for CoFeB/MgO in Fig. 6, which is specifically constructed by the *d*[001] occupation. The two important physical characteristics, PMA and TMR, are both influenced by the minority state of the *d*[001] occupation, as marked by the yellow part of the diagram. The *d*[001] minority states become more filled by thinning the CoFeB, and stronger PMA is enabled by *L*_*z*_ stabilization. In contrast, upon increasing thickness, the lift on *d*[001] minority states would decrease the occupation of electrons, which enhances the spin polarization by enlarging the difference between the minority (yellow) and immovable majority (blue) states. This polarization enhancement therefore gives rise to an effective regulation of TMR.

Nevertheless, it is essential to know that although PMA and TMR share the *d*[001] occupation, the two physical characteristics compete with each other because of the opposite thickness/broken-symmetry effects. This is confirmed by both macroscopic (Figs 1(a) and 5(b–d)) and microscopic (Fig. 5(a)) approaches. This actually explains the stagnant status of current MTJ technology, i.e., on promoting PMA for faster magnetization switching by reducing thickness, TMR is inevitably sacrificed owing to the submerged minority tunneling channel. This is why TMR appears much larger in an MTJ with in-plane anisotropy (IMA) than in that with PMA^10^,^11^,^12^. Therefore, recent research efforts devoted to improving the performance of the CoFeB/MgO MTJ could still stay in a scenario compromising the PMA–TMR competition. The concept of creating a sharp interface at the capping-layer/CoFeB junction to prevent inter-diffusion is essential, whereas limited, to improve the MTJ fundamentally. We suggest that the development of independent mechanisms of TMR and PMA is key to meet the demanded but contradictory requirement by turning the two physical characteristics into a complementary relationship. One possible alternative is to search for capping materials that can electronically/structurally drive the CoFeB’s PMA independently from MgO through a crystallization process, which of course has good contact resistance but poor miscibility with CoFeB to prevent the SPM formation. This also implies that current contact engineering in this particular material combination needs to be revisited. Through this finding, we hope to place future MTJ research on a more scientific footing and that hard work in contact engineering in this or similar MTJ systems will not go unrewarded. This study is also beneficial to the growing spintronic technology that requires a ferromagnet/semiconductor combination^20^,^21^, the inner workings of which between the ferromagnetic layer’s anisotropy and spin polarization are critical to the device’s functionality. Subtle modifications of these inner workings can be clearly resolved, as in the work presented here.

## Methods

Stacks consisting of Ta (30 Å)/CoFeB (*t* = 9, 10.5, 12, 13, 14, and 15 Å)/MgO (11 Å)/Ta (100 Å)/Si were deposited in a custom vacuum chamber with a base pressure of 10^−9^ Torr. Samples were denoted CFB*t* to correspond to the CoFeB film with a specific thickness (*t*). Metallic layers were deposited by dc magnetron sputtering (ANELVA C-7100) in an Ar atmosphere of 5 × 10^−3^ Torr with deposition rates of 1.25 Å/s and 0.5 Å/s for Ta and CoFeB, respectively. MgO layers were deposited by rf magnetron sputtering in a 5 × 10^−3^ Torr Ar atmosphere with a deposition rate of 0.089 Å/s. Upon deposition, the stacks were subjected to annealing at 300°C for 30 min. A transmission electron microscope (TEM) was used to probe the layer thickness and to confirm the [001]-textured MgO. Magnetic properties (hysteresis) were analyzed using a vibrating sample magnetometer (VSM) at desired temperatures. The TMR measurements were performed on the MTJ cells with a CoFeB-free layer of 10, 10.5, 11, and 13 Å, where the bottom CoFeB electrode was fixed at 9 Å. The MTJ cell was 180 nm in diameter and was microfabricated by photolithography and Ar ion milling, and TMR was collected using a Princeton Measurements Corporation – MicroMag 3900 transport measurement system, supported by a Keithley 2400 meter and a Kepco BOP 12/36 power supply. TMR was measured using a four-point probe for a magnetic-field range of + /- 1500 Oe. Sample fabrication and transport measurements were performed at the Electronics and Optoelectronics Research Laboratories, Industrial Technology Research Institute, Taiwan. For x-ray characterizations, x-ray absorption spectra (XAS) and x-ray magnetic circular dichroism (XMCD) were collected over Co/Fe *L*_*2*_/*L*_*3*_-edges to provide element-specific, spin-dependent electronic information. All the presented XAS and XMCD data were normalized to the post-edge jump and XAS integration, respectively, which ensured a reliable quantitative comparison by regularizing the data with respect to the variations in absorber concentration and any other aspects of the measurement. Sum-rule analyses^22^ were operated over the XAS/XMCD spectra to obtain atomic spin (*S*_*z*_) and orbital (*L*_*z*_) moments. All synchrotron data were collected at the National Synchrotron Radiation Research Center (NSRRC), Taiwan, under a magnetic field of 1 T.

## Additional Information

**How to cite this article**: Yang, C.-Y. *et al.* Competing Anisotropy-Tunneling Correlation of the CoFeB/MgO Perpendicular Magnetic Tunnel Junction: An Electronic Approach. *Sci. Rep.* **5**, 17169; doi: 10.1038/srep17169 (2015).

## Supplementary Material

Supplementary Information
