# Supplementary material for: Competing Anisotropy-Tunneling Correlation of the CoFeB/MgO Perpendicular Magnetic Tunnel Junction: An Electronic Approach
Source: Sci Rep. 2015 Nov 24;5:17169. doi: 10.1038/srep17169 (PMC4657009; doi:10.1038/srep17169)
Supplement: Supplementary Information [file srep17169-s1.pdf]

Supplementary materials

Competing Anisotropy-Tunneling Correlation of the CoFeB/MgO Perpendicular Magnetic Tunnel Junction: An Electronic Approach

Chao-Yao Yang,[1] Shu-Jui Chang,[1] Min-Han Lee,[2] Kuei-Hung Shen,[3] Shan-Yi Yang[3], Horng-Ji Lin,[4] and Yuan-Chieh Tseng[1,*]

[1]Department of Materials Science & Engineering, National Chiao Tung University, 1001 Ta Hsueh Road, Hsinchu, 30010, Taiwan, R.O.C.
[2]Undergraduate Honors Program of Nano Science and Engineering, National Chiao Tung University, 1001 Ta Hsueh Road, Hsinchu, 30010, Taiwan, R.O.C.
[3]Electronics and Optoelectronics Research Laboratories, Industrial Technology Research Institute Hsin-Chu, Taiwan 30010, R.O.C.
[4]National Synchrotron Radiation Research Center, Taiwan, 101 Hsin Ann Road, Hsinchu Science Park, Hsinchu 30076, Taiwan, R.O.C.
*yctseng21@mail.nctu.edu.tw

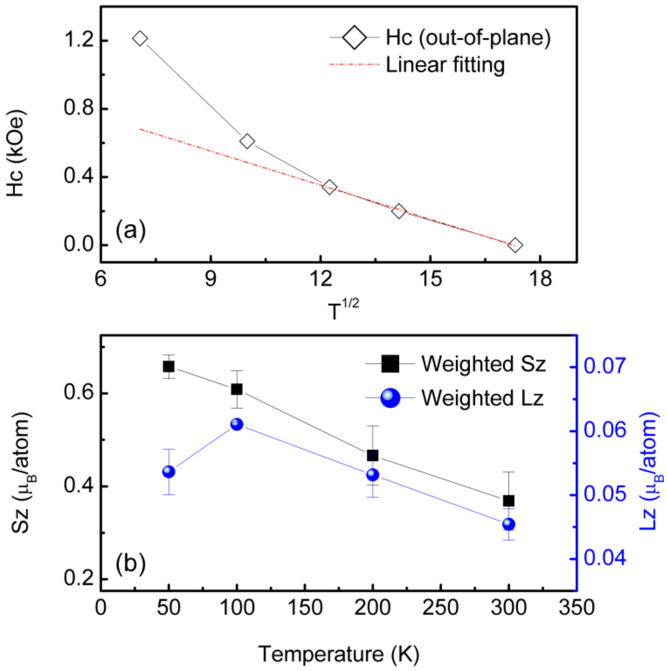

Figure 1 (a) H_c (out-of-plane) versus $T^{1/2}$, for CFB10.5 sample. The linear fitting (red line) is gained from the formula below, which is quoted from Ref. [1]. (b) Temperature-dependent, sum-rule analysis of weighted S_z (spin) and L_z (orbital) moments gained by weighting Co and Fe ratio in the CoFeB sample (Co:Fe=2:6). Data are for CFB10.5 sample.

$$H_{ci} = \frac{2K}{M_s}\left[1 - \left(\frac{25kT}{KV}\right)^{1/2}\right].$$

H_{ci} is the intrinsic H_c of the materials. K and Ms stand for the anisotropy constant and saturation

magnetization, respectively, which are both constants in this case. k and V are the Plank constant and the volume of a magnetic cluster, respectively. For a single magnetic phase, the H_c should appear in a linear dependency with $T^{1/2}$, but this is not the case seen here. Besides, Fig. (b) suggests a quenched L_z likely arising from a magnetic phase decoupling at low temperature (<100). Based on the two facts, we believe that the CFB10.5 is of a mixed-phase; namely, dead and alive phase.

Ref. [1] B. D. Cullity and C. D. Graham, Introduction to Magnetic Materials (John Wiley & Sons, Inc., Hoboken, New Jersey, 2009), pp. 383–390.
